# Analysis on Incidence and Mortality Trends and Age–Period–Cohort of Breast Cancer in Chinese Women from 1990 to 2019

**DOI:** 10.3390/ijerph20010826

**Published:** 2023-01-01

**Authors:** Meng Yin, Fang Wang, Yunquan Zhang, Runtang Meng, Xiaomei Yuan, Qun Wang, Yong Yu

**Affiliations:** 1 School of Public Health, Hubei University of Medicine, Shiyan 442000, China; 2 Department of Biostatistics, School of Public Health, Xuzhou Medical University, Xuzhou 221004, China; 3 Department of Epidemiology and Biostatistics, School of Public Health, Wuhan University of Science and Technology, Wuhan 430081, China; 4 Center of Health Administration and Development Studies, Hubei University of Medicine, Shiyan 442000, China; 5 School of Public Health, Hangzhou Normal University, Hangzhou 311121, China

**Keywords:** breast cancer, incidence, mortality, age–period–cohort model

## Abstract

Aims: To analyze the incidence and mortality trends of breast cancer among women in China from 1990 to 2019 and explore the effects of age, period, and cohort on the incidence and mortality of breast cancer. Methods: We performed a Joinpoint regression model to describe trends in breast cancer incidence and mortality. We used an age–period–cohort analysis model to estimate the impact of age, period, and cohort on breast cancer incidence and mortality. We collected breast cancer incidence and mortality among women aged 20–89 in China (1990–2019) from the Global Health Data Exchange (GHDx) database. Results: The crude incidence and mortality of breast cancer from 1990 to 2019 in Chinese women showed an increasing trend, with an average annual increase percentage (AAPC) of 4.69% and 2.18%, respectively. The analysis on the age–period–cohort model revealed that the risk of incidence increased first and then decreased with age and peaked at 55–59 years old, whereas the risk of mortality increased by approximately 60.34 times from 20 to 89 years old. The risk of incidence and mortality increased by 2.64 and 1.49 times with the passage of time, respectively. The later the birth cohort is, the lower the risk of incidence and mortality will be. Conclusion: From 1990 to 2019, the incidence and mortality of breast cancer among Chinese women showed an increasing trend, and the prevention and control situation of breast cancer was still grim. Therefore, visual examination and palpation examination should be actively carried out in adult women with breast cancer, and the conventional population after 40 years of age, the high-risk population carrying hereditary breast cancer gene and the elderly population should be assisted with imaging examination along with palpation examination. When treating patients suffering from breast cancer, in order to reduce the death rate, a personalized treatment plan should be developed based on the characteristics of different patients.

## 1. Introduction

Being the most common cancer among women, breast cancer cases have exceeded the number of lung cancer cases worldwide making it the leading type of cancer, with about 2.3 million new cases according to the 2020 Global Burden of Cancer Report released by the International Agency for Research on Cancer (IARC) of the World Health Organization (WHO). In 2020, breast cancer caused about 685,000 deaths worldwide, and it dominantly led to the death of females with cancer [1]. From 2008 to 2018, the growth rate of new breast cancer cases in the world remained above 30% [2]. Breast cancer is the main cancer among women in developed countries, and its burden in many regions with rapid economic development is also increasing [3]. In 2020, China had the highest incidence of breast cancer in the world, with approximately 120,000 deaths [4]. The incidence of breast cancer is always high, and the mortality rate is also increasing year by year, which has brought a serious burden of disease. The age–period–cohort model was used to investigate the effects of age, period, and cohort on the incidence and mortality of breast cancer in Chinese women from 1990 to 2019, aiming to provide evidence for the prevention and control of breast cancer. 

## 2. Materials and Methods

### 2.1. Data Source

The data were collected from the Global Health Data Exchange (GHDx), a project organized by the Institute for Health Measurement and Evaluation at the University of Washington [5]. The Chinese data for this study came from national and provincial disease surveillance centers, the China Statistical Yearbook, and published literature studies. The latest study estimated the disease burden from 369 cases and 87 risk factors for different age groups, genders, and causes in 204 countries and regions between 1990 and 2019 based on indicators such as incidence, mortality, years of life lost with premature death, years lived with disability, and disability-adjusted life years [6]. The web address for data acquisition of the GBD is http://ghdx.healthdata.org/gbd-results-tool (accessed on 27 March 2022). The data used in this study were the crude incidence and mortality of breast cancer in Chinese women from 1990 to 2019, and the incidence and mortality of the age group 20–89 years old (20–24, 25–29, …, 84–89 years).

### 2.2. Statistical Analysis

#### 2.2.1. Joinpoint Regression Model

The Joinpoint regression model is used in cancer epidemiology to describe the incidence and mortality trends of disease and identify significant turning points by conducting the Monte Carlo test. The Joinpoint regression model can be expressed as

(1) $$E\left[y|x\right]=\beta_{0}+\beta_{1}x+\delta_{1}{\left(x-\tau_{1}\right)}^{+}+\cdots +\delta_{k}{\left(x-\tau_{k}\right)}^{+}$$

Here, *y* is the incidence or mortality of breast cancer, which is the dependent variable, and *x* refers to the independent variable and usually represents the year. The constant term in the model is denoted by β. δ, τ, and k represent the regression coefficients of each piecewise function, the unknown turning points, and the number of turning points, respectively. When $\left(x-\tau_{1}\right)\geq 0$, ${\left(x-\tau_{k}\right)}^{+}=\left(x-\tau_{k}\right)$; otherwise, $\left(x-\tau_{k}\right)=0$.

In this study, the annual percent change (APC) and average annual percent change (AAPC) were used to analyze the incidence and mortality changes of breast cancer during the observation period. APC > 0 means that the incidence or mortality of breast cancer increases with time in the current period; APC < 0 denotes that the incidence or mortality of breast cancer decreases with time in the current period; APC = 0 represents that it does not change with time. Similarly, AAPC > 0 indicates that the incidence or mortality of breast cancer increases over time over the whole period; AAPC < 0 indicates that the incidence or mortality of breast cancer decreases over the whole period; AAPC = APC indicates that the incidence or mortality of breast cancer has a monotonically increasing or decreasing trend. The Joinpoint Regression Program 4.9.0.1 was used for Joinpoint regression analysis.

#### 2.2.2. Age–Period–Cohort Analysis

The age–period–cohort model is often used to describe the trend analysis of the incidence and mortality of chronic diseases, which can eliminate or control the interaction between age, cohort, and other factors to a certain extent [7]. The age–period–cohort model can be expressed as

(2) $$ln\left(Y_{xyz}\right)=\mu +\alpha_{x}+\beta_{y}+\gamma_{z}+\varepsilon_{xyz}$$

Here, $ln\left(Y_{xyz}\right)$ is the natural logarithm of the incidence and mortality of breast cancer in Chinese women, μ means the intercept of the regression equation, which is the baseline level of the risk of disease onset or death when age, period, and cohort factors are in effect, $\varepsilon_{xyz}$ represents the error term, and $\alpha_{x}$, $\beta_{y}$ and $\gamma_{z}$ are the effects of the parameters of age group, period group, and cohort group x, y and z, respectively, where x, y, z=1, 2, ….

In this paper, the endogenous factor method was applied to solve the problem of model unrecognition. This method was proposed by Fu [8] and Yang et al. [9], with the advantages of no prior model assumption and excellent results, and it can be directly calculated in Stata software.

Based on the endogenous factor method, the ages from 20–24 to 85–89 were divided into 14 age groups with consecutive five years as an age interval. The period from 1990 to 2019 was divided into six periods with five-year intervals. The corresponding 1905–1999 birth cohort was divided into 19 birth cohorts at consecutive five-year intervals.

Origin 2018 software was used to build a combined model of age, period, and cohort to fit the incidence and mortality of breast cancer. Stata15.0 software was used for age-age-cohort analysis, Akaike information criterion (AIC), and Bayesian information criterion (BIC) and variance were used to evaluate the degree of data-fitting diagnosis. The test level was 0.05.

## 3. Results

### 3.1. General Incidence and Mortality Trends of Breast Cancer

From 1990 to 2019, the incidence and mortality of breast cancer among Chinese women showed an increasing trend, with the crude incidence increasing from 14.14/100,000 in 1990 to 52.81/100,000 in 2019, and the crude mortality increasing from 7.22/100,000 in 1990 to 13.40/100,000 in 2019. The average annual growth rates were 4.69% and 2.18%, respectively. As shown in Table 1, the Joinpoint regression analysis on the incidence rate of breast cancer indicated that the incidence had three statistically significant turns from 1990 to 2019. From 2016 to 2019, the APC rate was 5.77% (*p* < 0.05), followed by 1995 to 2011 when the APC was 5.34% (*p* < 0.05). The Joinpoint regression analysis on the mortality rate of breast cancer suggested that it experienced five nodes of change between 1990 and 2019. The period of the highest growth rate was same as the incidence rate, and the APC was 3.92% (*p* < 0.01) during 2016–2019. From 2004 to 2007, the mortality rate of breast cancer increased the least, and the APC was 1.07% (*p* > 0.05), but the change was not statistically significant.

### 3.2. Age, Period, and Cohort Trends in Incidence and Mortality of Breast Cancer

#### 3.2.1. Age Trends

**Figure 1:**
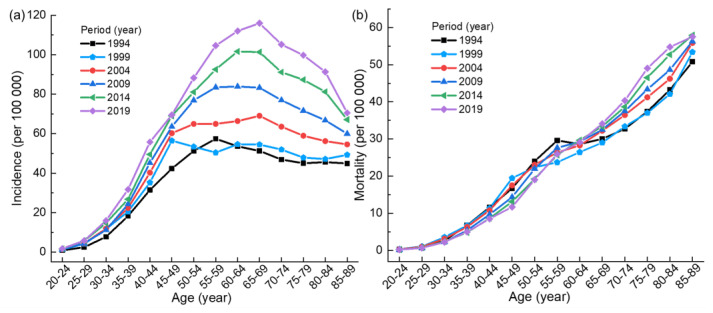
(**a**) Age trends of the incidence of breast cancer in Chinese women. (**b**) Age trends of the mortality of breast cancer in Chinese women.

Figure 1a shows that the incidence of breast cancer first increases and then decreases with the increase of age. In most periods, the incidence peaks in the 60–69 age group and then begins to decline. Among them, the incidence in 1994 (1999) is the highest in the 55–59 (45–49) age group. Figure 1b gives an increasing trend of mortality. The mortality in each period increases rapidly before the age group of 55–59, then slows down, and continues to accelerate after the age group of 65–69. Table S1 and Figure S1 show that the ratio of mortality and incidence in the same period presents an increasing trend with the increase of age. As can be seen from Figure S1, under the same incidence, the younger the age in each period, the lower the mortality rate.

#### 3.2.2. Period Trends

**Figure 2:**
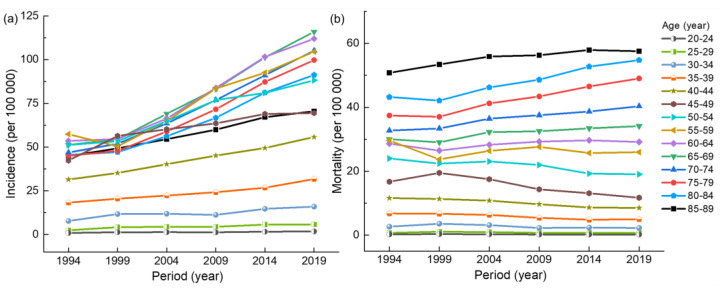
(**a**) Period trend of the incidence of breast cancer in Chinese women. (**b**) Period trend of the mortality breast of cancer in Chinese women.

As can be seen from Figure 2, the incidence and mortality of breast cancer in the 20–39 age group have no significant trend from 1990 to 2019, with less than 25 per 100,000 and 10 per 100,000, respectively. Figure 2a exhibits that the incidence of breast cancer in other age groups shows an overall upward trend with the passage of time, among which the 60–69 age group has the largest increase. In Figure 2b, the overall upward trend in mortality is not significant, with a downward trend in the 40–54 age group. Table S1 and Figure S1 illustrate that with the passage of time, the ratio of mortality and incidence shows a decreasing trend in all age groups. As can be seen in Figure S1, under the same incidence, the closer to modern times, the smaller the mortality is in each age group.

#### 3.2.3. Cohort Trends

**Figure 3:**
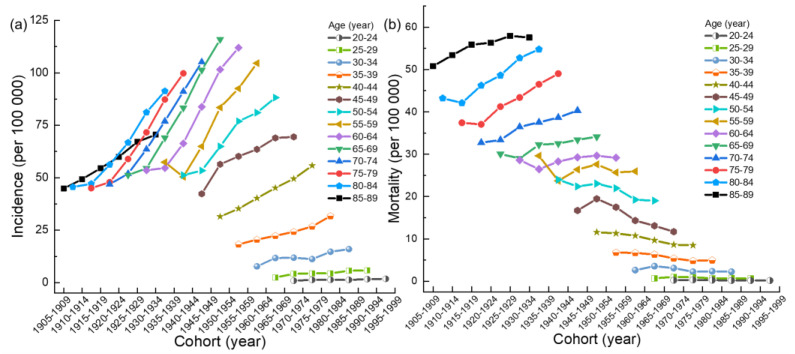
(**a**) Cohort trends of the incidence of breast cancer in Chinese women. (**b**) Cohort trends of the mortality of breast cancer in Chinese women.

According to Figure 3a, with the passage of the birth cohort, the overall age-specific incidence of breast cancer shows a rapid upward trend, and the incidence of breast cancer in the 55–59 age group shows a downward trend first and then an upward trend. In addition, the birth cohort has the least (greater) impact on the incidence of breast cancer in the 20–29 (60–84) age group. As shown in Figure 3b, the age-specific mortality of breast cancer first decreases and then increases, and the mortality of breast cancer in 30–34 and 45–49 years of age first increases and then decreases. Furthermore, age-specific mortality from breast cancer is least affected (greater) by the birth cohort in the 20–29 (75–84) age group.

### 3.3. The Results from Age–Period–Cohort Analysis on the Incidence and Mortality of Breast Cancer

#### 3.3.1. Age Effect

The results of age effect in Table 2 and Table 3 show that the incidence risk of breast cancer increases first and then decreases slowly with the increase of age, whereas the mortality risk increases with the increase of age. The effect coefficients of incidence and mortality risk are the lowest in the 20–24-year-old age group, with the lowest values of −2.68 and −3.06, respectively. The effect coefficient of incidence (mortality) risk is highest in the 55–59 (85–89) age group, with a value of 0.69 (1.04). The incidence (mortality) risk in the 55–59 (85–89) age group increases by 29.08 (60.34) times compared with that in the 20–24 age group.

#### 3.3.2. Period Effect

According to the period effect in Table 2 and Table 3, the incidence and mortality risk of breast cancer increases with the passage of time, and the increase of the effect coefficient of incidence is greater than that of mortality. The risk effect coefficients of incidence and mortality reached their highest values of 0.49 and 0.21 during 2015–2019, respectively. The risk of incidence and mortality increased by 2.64 and 1.49 times, respectively, compared with the lowest period.

#### 3.3.3. Cohort Effect

From 1905 to 1999, with the passage of birth cohort, the incidence and mortality risk of breast cancer shows a decreasing trend, as shown in Table 2 and Table 3. The risk effect coefficients of incidence and mortality decrease from 0.66 and 0.68 in 1905–1909 to −0.71 and −1.22 in 1995–1999, with a decrease of 207.58% and 279.41%, respectively.

## 4. Discussion

From 1990 to 2019, the crude incidence and mortality of breast cancer among women aged 20–89 in China showed an increased trend year by year, and the growth rate of the incidence was higher than the mortality, which confirmed that the current situation of female breast cancer in China was not optimistic, and China should strengthen the prevention and control of breast cancer.

From the perspective of age change trend, the incidence of breast cancer first increases and then decreases with the increase of age. Since 2009, the incidence rate of breast cancer in all age groups has increased, which may be related to the fact that more breast cancer cases have been detected since the implementation of the “Rural Women ‘Two Cancers’ Check Project Management Solutions” in 2009. The incidence peak appears in the 65–69 age group, which is about five years earlier than the high incidence rate of breast cancer in American women [10]. Among young and middle-aged women aged 30–55, the mortality begins to increase with age, because young patients with breast cancer have the characteristics of larger tumors, more lymph node involvement, higher recurrence rate, and worse prognosis [11]. Therefore, some studies suggested that Chinese women should receive breast cancer prevention knowledge education, especially those who are at high risk of carrying BRCA1 or BRCA2 germline mutations, as studies have shown that BRCA1/2 mutations can lead to hereditary breast cancer. Moreover, next-generation sequencing revealed that beyond BRCA1/2, mutations in homologous recombination effectors, such as PALB2, RAD51, ATM, BRIP1, BARD1, and CHEK2 also occur in breast cancer [12]. Chinese women should have regular breast examinations after they grow up. Families carrying BRCA1 or BRCA2 germline mutation genes and individuals over 25 years of age who have not yet been tested are encouraged to do so. The conventional population should be screened with imaging examination after the age of 40, and the high-risk population should be screened earlier. The screening method should be adjusted according to the characteristics of the breast in different periods of women, so as to improve the detection rate of breast cancer. Individuals who have a germline pathogenic variant in BRCA1 or BRCA2 are counseled at the time of disclosure of molecular genetic test results about their options for prevention of primary manifestations, such as consideration of prophylactic bilateral mastectomy and chemoprevention with tamoxifen to significantly reduce incidence and mortality [13,14]. After 65–69 years of age, the mortality continues to rise, and reaches its peak at 85–89 years old, which may be related to the decline of the body function of the elderly and their poor tolerance to various treatment methods, and seldom follows the treatment guidelines of elderly patients in treatment, thus resulting in the undertreatment or overtreatment [15]. In the same period, the ratio of mortality to incidence increases with age, which means that under the same incidence, the older the age, the higher the mortality. Risk factors such as increasing age and more underlying diseases make the treatment effect and prognosis of elderly female breast cancer patients poor, and the mortality of breast cancer will increase significantly. Therefore, individualized treatment should be developed based on the overall health status and tolerance of the elderly.

In general, the incidence of breast cancer continues to rise with the passage of time. Since 1999, the incidence has increased significantly, but this change becomes significant only at ages over 50 years, which may be related to the fact that China entered the aging society in 1999, and the aging population increased the number of elderly breast cancer patients. The mortality shows a clear downward trend in the 45–49 age group, which may be attributed to China’s increased breast cancer screening efforts in recent years [16,17]. Therefore, more breast cancer patients were detected, and early screening increased the incidence of breast cancer and reduced the mortality [18]. The ratio of mortality to incidence in the same age group has a declining trend over time, which means that under the same incidence, the closer to modern times, the lower the mortality. With the progress of medical technology, significant progress has been made in the research on effective treatment of breast cancer. In recent years, the application of newly developed drugs, targeted therapy, immunotherapy, and other technologies have gradually matured, promoting the development of personalized precision medicine, and thus reducing the mortality of breast cancer [19].

From the trend of birth cohort, the incidence, and mortality of young people in the birth cohort close to modern times are lower, whereas the morbidity and mortality of elderly people in the older birth cohort are higher, which is caused by the relationship between the birth cohort and period and age, so the age–period–cohort model is needed for further analysis.

The age–period–cohort trend of the incidence and mortality of breast cancer in Chinese females cannot eliminate the interaction among the three factors. In order to explore the independent effects of age, period, and cohort, it is necessary to use the age–period–cohort model for further analysis. The analysis results of the age–period–cohort model proved that in the age effect, the risk of breast cancer increases first and then decreases with the increase of age and reaches the peak in the 55–64 age group, which is similar to the results of other studies [20,21,22]. Whether this phenomenon is related to the decline of female fertility caused by the implementation of family planning in 1970 in China still needs further explanation [23]. The risk of the mortality of breast cancer in Chinese women increases with the increase of age, which indicates that the risk of mortality in elderly women is the highest. Therefore, more attention should be paid to elderly women with the highest risk of the mortality of breast cancer. The period effect shows that the risk of the incidence and mortality of breast cancer in Chinese women increased from 1990 to 2019. With the rapid development of social economy, people’s living standards have been greatly improved, and their lifestyle has gradually become Westernized. They are more inclined to be on a diet with high fat, high sugar, high protein, and low dietary fiber. Physical activity is gradually reduced, and the pace of life is gradually accelerated [24]. Unhealthy behaviors such as obesity, lack of exercise, stress, and irregular work and rest increase the risk of the incidence and mortality of breast cancer. Moreover, the changes in women’s reproductive patterns and attitudes in recent years, including late childbearing, fewer children, and shorter periods of breastfeeding, also increase the risk of breast cancer [25]. Therefore, women’s awareness of cancer prevention should be actively strengthened. Institutions such as schools and communities can popularize the knowledge of breast cancer prevention to women and urge women within the age range of breast cancer to undergo breast screening regularly. States and governments should introduce a series of policies to encourage women at the right age to bear children as soon as possible and advocate breastfeeding. Studies have shown that the increase in the number of births of Chinese women is related to the reduction of the risk of breast cancer in postmenopausal women [26]. In recent years, the impact of the implementation of the “two-child policy” and “three-child policy” on the incidence of breast cancer in Chinese women in the future is worthy of further research. In the trend of birth cohort changes, the cohort effect of the incidence and mortality of breast cancer on Chinese women has declined throughout the observation period, and women born late in the cohort have lower risk of incidence and mortality than women born early in the cohort. The lower risk may be associated with higher levels of education and health awareness among the younger generation [27]. In recent years, the rapid development and maturity of medical technology has made the treatment plan for breast cancer more individualized and accurate, which has greatly reduced the risk of the mortality of breast cancer.

## 5. Strengths and Limitations

There are some strengths and limitations in the present study. The strengths of this study are, first, the use of a large time span—nearly three decades of data—from the GBD database from 1990 to 2019. Secondly, the age range of the study population is 20 to 89-year-old Chinese women, which covers a wide range. Then, it used the age–period–cohort model to analyze the individual effects of age, period, and cohort, which makes up for the traditional descriptive analysis methods that cannot accurately reflect the impact of age, period, and cohort on incidence and mortality. The limitations of this study are first due to the limited nature of the data, which did not pay attention to the differences in incidence and mortality in different regions of China, as well as the differences in diagnosis and treatment caused by different levels of medical care. Secondly, it is difficult to completely avoid data inaccuracies due to data integrity and quality issues.

## 6. Conclusions

In conclusion, the incidence and mortality of breast cancer in China show an upward trend, and the prevention and control situation remains grim. We should strengthen the prevention and control of breast cancer in China, and call for women to accept regular breast palpation and visual examinations as soon as they become adults. Most importantly, it is recommended that the conventional population (after reaching 40 years of age) the high-risk population carrying hereditary breast cancer genes, and the elderly population should be combined with imageological examinations. At the same time, we should popularize the knowledge of breast cancer and improve the awareness of all women with regard to breast cancer prevention. For the treatment of breast cancer patients, in order to prolong the life of breast cancer patients and improve the quality of life, it is necessary to formulate personalized treatment plans based on the type of breast cancer, the health status of patients, and their own needs. In short, the disease burden of females with breast cancer in China is a major problem that needs to be solved now and in the future.

## Figures and Tables

**Table 1:** Crude incidence and mortality trends of breast cancer in Chinese women from 1990 to 2019.

Variable	Year	APC	95% CI	*p*	AAPC	95% CI	*p*
Incidence	1990–1995	3.42	2.85–4.01	<0.01			
	1995–2011	5.34	5.26–5.41	<0.01	4.69	4.47–4.91	<0.01
	2011–2016	3.26	2.58–3.93	<0.01			
	2016–2019	5.77	4.10–7.47	<0.01			
Mortality	1990–1997	1.43	1.21–1.65	<0.01			
	1997–2001	3.19	2.56–3.81	<0.01			
	2001–2004	2.34	1.09–3.61	<0.01			
	2004–2007	1.07	−0.06–2.22	0.06	2.18	1.96–2.39	<0.01
	2007–2016	2.05	1.91–2.19	<0.01			
	2016–2019	3.92	2.86–5.00	<0.01			

**Table 2:** Age-period-cohort model of the incidence of breast cancer in Chinese women from 1990 to 2019.

Age (Year)	Effect Coefficient	Standard Error	Z	*p*	95% CI	
					Lower	Upper
20–24	−2.68	0.34	−7.78	0.00	−3.36	−2.01
25–29	−1.60	0.19	−8.63	0.00	−1.96	−1.24
30–34	−0.70	0.13	−5.44	0.00	−0.95	−0.44
35–39	−0.11	0.10	−1.07	0.28	−0.31	0.09
40–44	0.38	0.09	4.41	0.00	0.21	0.55
45–49	0.63	0.08	8.29	0.00	0.48	0.78
50–54	0.68	0.07	9.89	0.00	0.55	0.81
55–59	0.69	0.06	11.07	0.00	0.57	0.81
60–64	0.67	0.06	11.76	0.00	0.56	0.79
65–69	0.64	0.05	11.87	0.00	0.53	0.75
70–74	0.52	0.05	9.72	0.00	0.42	0.63
75–79	0.42	0.06	7.60	0.00	0.31	0.53
80–84	0.31	0.06	5.24	0.00	0.19	0.43
85–89	0.13	0.07	1.98	0.05	0.00	0.27
Period (year)						
1990–1994	−0.48	0.05	−10.08	0.00	−0.57	−0.39
1995–1999	−0.34	0.04	−8.17	0.00	−0.42	−0.26
2000–2004	−0.11	0.04	−2.93	0.00	−0.18	−0.04
2005–2009	0.11	0.03	3.31	0.00	0.05	0.18
2010–2014	0.32	0.04	9.08	0.00	0.25	0.39
2015–2019	0.49	0.04	13.18	0.00	0.42	0.56
Cohort (year)						
1905–1909	0.66	0.15	4.29	0.00	0.36	0.96
1910–1914	0.56	0.11	4.98	0.00	0.34	0.77
1915–1919	0.42	0.09	4.51	0.00	0.24	0.60
1920–1924	0.33	0.08	4.07	0.00	0.17	0.48
1925–1929	0.28	0.07	3.88	0.00	0.14	0.42
1930–1934	0.23	0.07	3.58	0.00	0.11	0.36
1935–1939	0.23	0.07	3.47	0.00	0.10	0.36
1940–1944	0.17	0.07	2.44	0.01	0.03	0.31
1945–1949	0.14	0.08	1.90	0.06	0.00	0.29
1950–1954	0.14	0.08	1.73	0.08	−0.02	0.30
1955–1959	0.05	0.09	0.60	0.55	−0.12	0.22
1960–1964	−0.06	0.10	−0.67	0.50	−0.25	0.12
1965–1969	−0.18	0.11	−1.72	0.09	−0.39	0.03
1970–1974	−0.32	0.12	−2.72	0.01	−0.55	−0.09
1975–1979	−0.37	0.13	−2.82	0.01	−0.63	−0.11
1980–1984	−0.42	0.16	−2.67	0.01	−0.73	−0.11
1985–1989	−0.51	0.21	−2.43	0.02	−0.92	−0.10
1990–1994	−0.62	0.34	−1.82	0.07	−1.29	0.05
1995–1999	−0.72	0.78	−0.92	0.36	−2.25	0.81
Intercept	3.49	0.05	71.36	0.00	3.39	3.59
Variance	7.48					
Akaike information criterion (AIC)	6.30					
Bayesian information criterion (BIC)	−205.20					

**Table 3:** Age–period–cohort model of the mortality of breast cancer in Chinese women from 1990 to 2019.

Age (Year)	Effect Coefficient	Standard Error	Z	*p*	95% CI	
					Lower	Upper
20–24	−3.06	0.79	−3.87	0.00	−4.60	−1.51
25–29	−2.00	0.42	−4.70	0.00	−2.83	−1.17
30–34	−0.95	0.27	−3.48	0.00	−1.49	−0.42
35–39	−0.35	0.22	−1.61	0.11	−0.77	0.08
40–44	0.04	0.18	0.21	0.83	−0.32	0.40
45–49	0.33	0.16	2.10	0.04	0.02	0.63
50–54	0.53	0.13	3.99	0.00	0.27	0.79
55–59	0.62	0.11	5.44	0.00	0.40	0.85
60–64	0.61	0.10	6.01	0.00	0.41	0.81
65–69	0.66	0.09	7.06	0.00	0.48	0.84
70–74	0.75	0.09	8.29	0.00	0.57	0.92
75–79	0.85	0.09	9.03	0.00	0.67	1.04
80–84	0.93	0.11	8.76	0.00	0.72	1.14
85–89	1.04	0.12	8.35	0.00	0.79	1.28
Period (year)						
1990–1994	−0.19	0.08	−2.30	0.02	−0.35	−0.03
1995–1999	−0.15	0.06	−2.37	0.02	−0.28	−0.03
2000–2004	−0.03	0.05	−0.57	0.57	−0.13	0.07
2005–2009	0.04	0.05	0.69	0.49	−0.07	0.14
2010–2014	0.12	0.06	1.90	0.06	0.00	0.24
2015–2019	0.21	0.08	2.80	0.01	0.06	0.36
Cohort (year)						
1905–1909	0.68	0.21	3.20	0.00	0.26	1.10
1910–1914	0.66	0.18	3.78	0.00	0.32	1.01
1915–1919	0.58	0.16	3.75	0.00	0.28	0.89
1920–1924	0.54	0.14	3.75	0.00	0.26	0.82
1925–1929	0.51	0.14	3.77	0.00	0.25	0.78
1930–1934	0.47	0.13	3.54	0.00	0.21	0.73
1935–1939	0.46	0.14	3.27	0.00	0.19	0.74
1940–1944	0.39	0.15	2.52	0.01	0.09	0.69
1945–1949	0.32	0.17	1.88	0.06	−0.01	0.65
1950–1954	0.27	0.19	1.44	0.15	−0.10	0.63
1955–1959	0.13	0.21	0.62	0.53	−0.27	0.53
1960–1964	−0.05	0.23	−0.22	0.83	−0.49	0.40
1965–1969	−0.21	0.25	−0.84	0.40	−0.71	0.28
1970–1974	−0.41	0.28	−1.46	0.15	−0.97	0.14
1975–1979	−0.55	0.33	−1.67	0.09	−1.19	0.09
1980–1984	−0.68	0.40	−1.71	0.09	−1.47	0.10
1985–1989	−0.85	0.55	−1.53	0.13	−1.94	0.24
1990–1994	−1.04	0.97	−1.07	0.28	−2.94	0.86
1995–1999	−1.22	2.30	−0.53	0.60	−5.72	3.28
Intercept	2.40	0.14	17.08	0.00	2.12	2.67
Variance	2.96					
Akaike information criterion (AIC)	5.26					
Bayesian information criterion (BIC)	−209.71					

## Data Availability

The datasets analysed during the current study are available in the publicly available datasets. This data can be found here: http://ghdx.healthdata.org/gbd-results-tool (access on 27 March 2022).

## Supplementary Materials

The following supporting information can be downloaded at: https://www.mdpi.com/article/10.3390/ijerph20010826/s1, Figure S1: A figure of breast cancer mortality/incidence parameters for Chinese women by age and period was obtained; Table S1: A table of breast cancer mortality/incidence parameters for Chinese women by age and period was obtained.
