# Supplementary material for: Analysis on Incidence and Mortality Trends and Age–Period–Cohort of Breast Cancer in Chinese Women from 1990 to 2019
Source: Int J Environ Res Public Health. 2023 Jan 1;20(1):826. doi: 10.3390/ijerph20010826 (PMC9820148; doi:10.3390/ijerph20010826)

International Journal of
Environmental Research and Public Health

MDPI

Supporting Information

Analysis on Incidence and Mortality Trends and Age–Period–Cohort of Breast Cancer in Chinese Women from 1990 to 2019

Meng Yin, Fang Wang, Yunquan Zhang *, Runtang Meng, Xiaomei Yuan, Qun Wang, Yong Yu *

Table S1

A table of breast cancer mortality/incidence parameters for Chinese women by age and period was obtained.

Age(Year)	Mortality/Incidence (Ratio)					
	1990-1994	1995-1999	2000-2004	2005-2009	2010-2014	2015-2019
20-24	0.272	0.239	0.196	0.144	0.122	0.106
25-29	0.287	0.252	0.216	0.160	0.130	0.114
30-34	0.339	0.304	0.262	0.201	0.160	0.141
35-39	0.371	0.327	0.283	0.223	0.180	0.156
40-44	0.368	0.321	0.268	0.214	0.175	0.153
45-49	0.395	0.345	0.290	0.226	0.190	0.168
50-54	0.468	0.419	0.355	0.285	0.238	0.216
55-59	0.516	0.470	0.406	0.331	0.278	0.248
60-64	0.534	0.484	0.426	0.349	0.292	0.260
65-69	0.586	0.532	0.467	0.390	0.330	0.294
70-74	0.698	0.642	0.573	0.488	0.424	0.383
75-79	0.831	0.773	0.699	0.606	0.533	0.491
80-84	0.948	0.893	0.822	0.728	0.649	0.600
85-89	1.130	1.083	1.024	0.939	0.863	0.816

Figure S1

A figure of breast cancer mortality/incidence parameters for Chinese women by age and period was obtained.

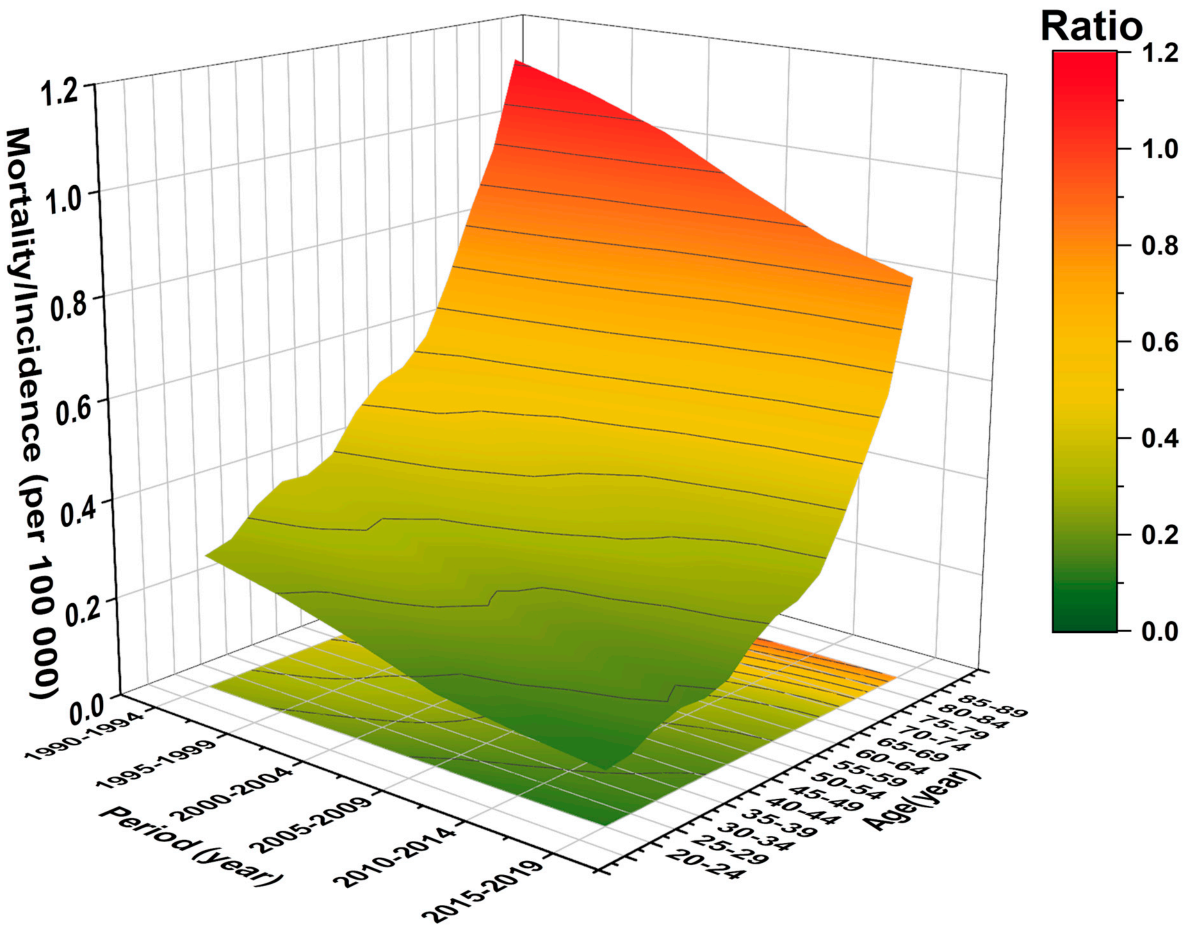

Supplement: Supplementary file 1 [file ijerph-20-00826-s001.zip › ijerph-2054271-supplementary.pdf]
